# *Ginkgo biloba* in the Aging Process: A Narrative Review

**DOI:** 10.3390/antiox11030525

**Published:** 2022-03-09

**Authors:** Sandra Maria Barbalho, Rosa Direito, Lucas Fornari Laurindo, Ledyane Taynara Marton, Elen Landgraf Guiguer, Ricardo de Alvares Goulart, Ricardo José Tofano, Antonely C. A. Carvalho, Uri Adrian Prync Flato, Viviane Alessandra Capelluppi Tofano, Cláudia Rucco Penteado Detregiachi, Patrícia C. Santos Bueno, Raul S. J. Girio, Adriano Cressoni Araújo

**Affiliations:** 1 Postgraduate Program in Structural and Functional Interactions in Rehabilitation, University of Marília (UNIMAR), Marília 17525-902, SP, Brazil; eleng@unimar.br (E.L.G.); rgoulart@unimar.br (R.d.A.G.); ricardo.tofano@unimar.br (R.J.T.); antonely.prefeito@ibaiti.pr.gov.br (A.C.A.C.); uriflato@unimar.br (U.A.P.F.); claudia.detregiachi@unimar.br (C.R.P.D.); patriciabueno@unimar.br (P.C.S.B.); araujo.01@unimar.br (A.C.A.); 2 Department of Biochemistry and Pharmacology, School of Medicine, University of Marília (UNIMAR), Avenida Higino Muzzi Filho, 1001, Marília 17525-902, SP, Brazil; 1851730@unimar.br (L.F.L.); 1813975@unimar.br (L.T.M.); viviane.tofano@unimar.br (V.A.C.T.); 3 School of Food and Technology of Marilia (FATEC), Avenida Castro Alves, Marília 17500-000, SP, Brazil; 4 Laboratory of Systems Integration Pharmacology, Clinical & Regulatory Science, Research Institute for Medicines (iMed.ULisboa), Faculdade de Farmácia, Universidade de Lisboa, Av. Prof. Gama Pinto, 1649-003 Lisbon, Portugal; rdireito@ff.ulisboa.pt; 5 Department of Animal Sciences, School of Veterinary Medicine, University of Marília (UNIMAR), Avenida Higino Muzzi Filho 1001, Marília 17525-902, SP, Brazil; rgirio@unimar.br

**Keywords:** *Gingko biloba*, aging, neurodegenerative diseases, Alzheimer’s disease, metabolic syndrome, cardiovascular disease, cancer

## Abstract

Neurodegenerative diseases, cardiovascular disease (CVD), hypertension, insulin resistance, cancer, and other degenerative processes commonly appear with aging. *Ginkgo biloba* (GB) is associated with several health benefits, including memory and cognitive improvement, in Alzheimer’s disease (AD), Parkinson’s disease (PD), and cancer. Its antiapoptotic, antioxidant, and anti-inflammatory actions have effects on cognition and other conditions associated with aging-related processes, such as insulin resistance, hypertension, and cardiovascular conditions. The aim of this study was to perform a narrative review of the effects of GB in some age-related conditions, such as neurodegenerative diseases, CVD, and cancer. PubMed, Cochrane, and Embase databases were searched, and the PRISMA guidelines were applied. Fourteen clinical trials were selected; the studies showed that GB can improve memory, cognition, memory scores, psychopathology, and the quality of life of patients. Moreover, it can improve cerebral blood flow supply, executive function, attention/concentration, non-verbal memory, and mood, and decrease stress, fasting serum glucose, glycated hemoglobin, insulin levels, body mass index, waist circumference, biomarkers of oxidative stress, the stability and progression of atherosclerotic plaques, and inflammation. Therefore, it is possible to conclude that the use of GB can provide benefits in the prevention and treatment of aging-related conditions.

## 1. Introduction

The medicine associated with basic sanitation and lifestyle modifications has benefited the world’s population, and the repercussions are seen as an increase in longevity. On the other hand, the aging process is associated with physical and functional changes in different tissues and organs and is related to genetic factors, mutations, telomere loss, oxidative stress, mitochondrial dysfunction, inflammation, immune disorders, and other modifications that interfere with homeostasis. Neurodegenerative diseases, cardiovascular disease (CVD), hypertension, insulin resistance, cancer, osteoporosis, and other degenerative processes may commonly appear with aging, leading to a significant burden on health care systems [1,2,3].

Drug therapy can prevent age-related conditions or can be used in the therapeutic approach. However, the high cost of many medications, their side effects, and their response refractoriness in many patients makes other alternatives worth seeking. For these reasons, herbal medicine has been considered for maintaining health or as a therapeutic approach for aging people. Herbal therapies are relevant since they show few side effects, present cultural acceptability, and have reduced costs. These therapies can contribute to well-being and the prevention of several aging conditions and chronic illnesses. Among the medicinal plants that can improve age-related issues is *Gingko biloba* (GB) [4,5,6], a medicinal plant belonging to the *Ginkgoaceae* family in Ginkgoopsida that is considered to be the oldest tree alive in the world (*Ginkgo* species are from the Permian Period, around 286–248 million years ago). The gray-colored bark tree is native to China, Japan, and Korea; however, it is distributed in many regions of Europe, America, India, and New Zealand [7,8,9].

**Figure 1:**
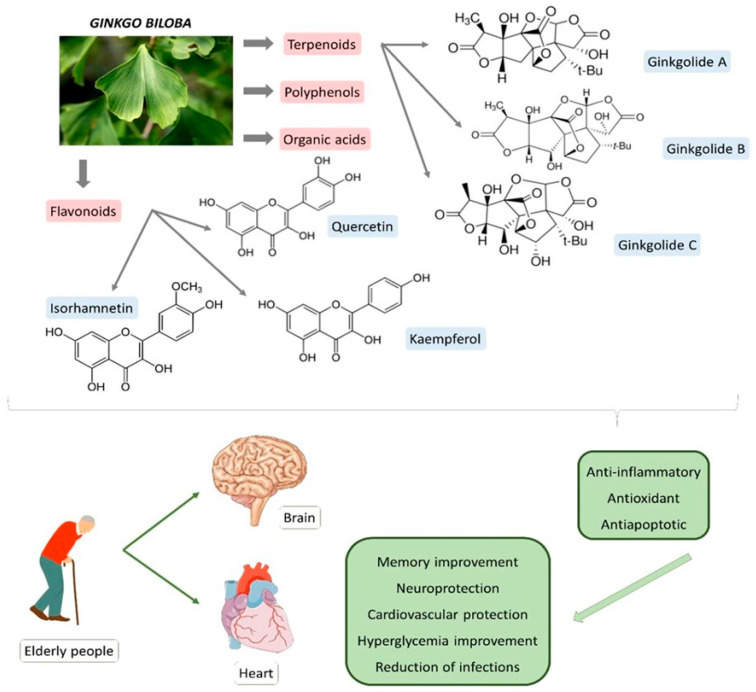
Primary bioactive compounds of *Ginkgo biloba* and its effects.

GB has been used for medicinal purposes for centuries. In traditional medicine, it is primarily used for respiratory and cardiovascular conditions; in Chinese medicine, it has been considered for treating pulmonary issues, bladder infections, and alcohol abuse. The therapeutic potential is attributed to its bioactive compounds that are mainly represented by terpenoids, flavonoids, polyphenols, and organic acids. The primary terpenoids are ginkgolides. GB standard extract contains 2.6 to 3.2% bilobalide, 2.8 to 3.4% ginkgolides (A, B, and C), and 24% flavone glycosides (quercetin, isorhamnetin, and kaempferol) [10,11,12,13]. Figure 1 summarizes the primary bioactive compounds of GB and its effects.

The leaves and the seeds of GB may represent one of the most common phytopharmaceutical products in the United States and Europe. GB is also commercialized as an extract (EGB 761^®^), producing several health benefits for memory, cognition, Alzheimer’s disease (AD), Parkinson’s disease (PD), and dementia. These pharmacological effects are attributed to the antiapoptotic, antioxidant, and anti-inflammatory actions that, in addition to having effects on cognition, also exert benefits in other conditions associated to aging-related processes, such as insulin resistance, hypertension, dyslipidemia, and cardiovascular disorders. In many European states, EGB 761^®^ is the only drug therapy in the guideline for the treatment of mild cognitive impairment (MCI) [9,10,14,15,16].

Based on the above beneficial effects, this study aimed to perform a review on the effects of GB on some age-related conditions, such as neurodegenerative diseases, metabolic syndrome, and CVD.

## 2. Materials and Methods

### 2.1. Focused Question

The focal question of this review was: What are the effects of *Ginkgo biloba* on the aging process?

### 2.2. Language

Only studies in English were selected for this review.

### 2.3. Databases

The PubMed, Embase and Cochrane databases were searched. The descriptors used were *Ginkgo biloba* or *Ginkgo biloba* extract and neurodegenerative diseases, or memory, or Alzheimer’s disease, or Parkinson’s disease, or dementia, or hypertension, or insulin resistance, or metabolic syndrome, or cardiovascular diseases. These descriptors helped identify studies related to *Ginkgo biloba* and some aspects of the aging process. Although this is a narrative review, we followed the PRISMA (Preferred Reporting Items for a Systematic Review and Meta-Analysis) guidelines for improving the strategy of paper screening [17].

### 2.4. Study Selection

This review included studies that reported *Ginkgo biloba* or *Ginkgo biloba* extract to treat disorders associated with the aging process. The inclusion criteria comprised randomized clinical trials (RCTs), double-blind, and placebo-controlled studies that reported the use of GB in patients over the age of 45 years. We only included full texts. The PICO (population, intervention, comparison, and outcomes) format was followed to build this review.

The exclusion criteria were in vitro studies, studies with animals, clinical trials that associated different herb formulations, reviews, studies not in English, poster presentations, case reports, and editorials. Reviews were consulted to help in the discussion section but were not included in the systematization of the data.

### 2.5. Data Extraction

The search period for this review included the past ten years (January 2011 to May 2021). The selected studies are shown in Table 1.

### 2.6. Quality Assessment

The bias risk evaluation was performed by selecting the study, detection, and reporting bias of each clinical trial. Other risks of bias in the selection of patients, classification of interventions, evaluation of outcomes, and missing data were also considered. The Cochrane Handbook for Systematic Reviews of Interventions was used to perform the quality assessment [18].

**Table 1:** The effects of *Ginkgo biloba* on neurodegenerative diseases, diabetes, and metabolic syndrome.

Reference	Local	Model and Patients	Intervention	Outcomes	Adverse Effects (AEs)
**Neurodegenerative Diseases**					
[19]	Germany	Randomized, double-blind, placebo-controlled, mono-center trial with 188 mentally healthy male and female subjects, 45–65 y, with higher secondary education.	Subjects received EGb 761 240 mg/day or placebo/6 w.	Subjects treated with EGb 761 significantly improved the number of appointments correctly recalled. The effects on qualitative recall performance (proportion of false to correct items) were similar. GB had no superiority in another routine memory test that required recognition of a driving route.	Seven AEs in the EGb 761 group (headache, *n* = 4; gastric complaints, *n* = 3) and five in the placebo group (gastric complaints, *n* = 3; conjunctivitis, nettle rash). No serious AEs occurred during the study.
[20]	Ukraine	Randomized, double-blind, placebo-controlled, multicenter trial with 410 outpatients (132 male and 272 female), 50 y or older with mild to moderate dementia (AD, vascular dementia, or mixed form) with scores between 9 and 23 on the SKT cognitive tests battery, at least 5 in the NPI and 3 or more in at least one item of the NPI.	Patients were allocated to receive 240 mg of EGb 761 or placebo once a day/24 w. Primary efficacy measures were the SKT, and the 12-item NPI.	EGb was found to be significantly superior to placebo in the treatment of patients with neuropsychiatric symptoms; significant improvement on the SKT and NPI total score (placebo showed deterioration on SKT).	AE rates were similar for both treatment groups (headache, respiratory tract infection, dizziness, angina pectoris, diarrhea, and tinnitus).
[21]	Republic of Belarus, Republic of Moldova, and Russian Federation	Multicenter, double-blind, randomized, placebo-controlled study with 410 outpatients, ≥50 y (279 female and 123 male) with mild to moderate dementia (AD or vascular dementia) related to neuropsychiatric symptoms. Participants scored 9–23 on the SKT cognitive battery and at least 6 on the NPI, with at least one of four items rated at minimum 4.	Patients were randomized to receive 240 mg of EGb 761 once a day/24 w.	Treatment with EGb 761 was safe and significantly improved functional measures, cognition, psychopathology, and quality of life of patients.	Lethal cardiac arrest due to chronic heart failure in a patient suffering from multiple illnesses; a lethal ischaemic infarction in the region of the terminal branches of the middle and posterior cerebral arteries in a patient with a history of DM, hypertension, atherosclerosis, myocardial infarction, and a previous stroke.
[22]	Iran	Randomized double-blind study with 56 patients (23 male and 28 female), 50–75 y, with a diagnosis of probable AD according to the DSM IV.	Patients were allocated into (1) GB (120 mg once a day) or (2) rivastigmine (4.5 mg once a day)/24 w. The SMT and the MMSE measured the severity of dementia.	There was a significant improvement of the MMSE scores in the rivastigmine group but not in the GB group. The same results were observed for the SMT.	AEs were not reported.
[23]	China	Randomized clinical trial with 80 patients with VCIND (60–75 y, 46 males and 34 females), disorder shown by revised mini-mental state examination.	Group 1 received 75 mg aspirin 3 times/d/3 m. Group 2 received 19.2 mg GBT 3 times/d/3 m with anti-platelet aggregation drugs. MoCA and TCD were used to observe changes in cognitive ability and cerebral blood flow in VCIND patients.	GBT can improve the therapeutic efficacy and enhance the cognitive ability and cerebral blood flow supply of patients with VCIND.	AEs were not reported.
[24]	EUA	Open-label phase II clinical trial with 34 patients (23 female and 11 male), symptomatic irradiated brain tumor survivors, life expectancy ≥ 30 w, partial or whole-brain radiation ≥ 6 m before enrollment, no imaging evidence of tumor progression in previous 3 m, or stable or decreasing steroid dose, and no brain tumor treatment planned while in the study.	The GB dose was 120 mg/day (40 mg t.i.d.) for 24 w, followed by a 6 w washout period.	There were significant improvements at 24 w in executive function, attention/concentration, and non-verbal memory and mood.	AEs included gastrointestinal toxicity and intracranial hemorrhage.
[25]	Russia	Double-blind randomized multicenter trial with 160 patients (124 female and 33 male, ≥55 y) with MCI who scored at least 6 on the 12-item NPI were enrolled.	Patients received 240 mg of EGb 761 daily or placebo/24 w. Effects on NPS were evaluated using the NPI, the state subscore of the State-Trait Anxiety Inventory and the Geriatric Depression Scale.	EGb 761 ameliorated NPS and cognitive performance in subjects with MCI. The drug was safe and well tolerated.	Headache, increased blood pressure, respiratory tract infection, and dyspepsia/epigastric discomfort.
[26]	China	Randomized clinical trial with 80 cerebral infarction patients (46 males and 34 females) 60–75 y.	Group 1 received aspirin 75 mg 3 times/d/3 m, and Group 2 received 40 m of GBT with aspirin 3 times/d/3 m.	GBT improved the therapeutic efficacy, cerebral blood flow supply, and cognitive ability of patients with VCIND.	AEs were not reported.
[27]	Germany	Randomized double-blind placebo-controlled trial with 75 volunteers (50–65 y) with subjective memory impairment evidenced by at least one answered item as “rather often” or “very often” or at least five questions answered “sometimes” in the Prospective and Retrospective Memory Questionnaire.	240 mg EGb 761^®^ or placebo once a day in the morning as film coated tablet/56 ± 4 days.	Baseline fMRI data evidenced BOLD responses in regions commonly activated by the specific tasks. Task-switch costs reduced with EGb761^®^, suggesting improvement in cognitive flexibility. Go–NoGo task reaction times corrected for error rates showed a trend of improved response inhibition.	Headache.
[28]	Germany	Randomized, double-blind, placebo-controlled exploratory study with 50 patients (25 female and 25 males; 50–85 y) with MCI and associated dual task-related gait impairment.	Patients received GBE (Symfona^®^ forte 120 mg) 2 times/d/6 m or placebo capsules. A 6 m open-label phase with identical GBE dosage followed. Gait was quantified at months 0, 3, 6, and 12.	After 6 m, dual task-related cadence increased in the intervention group compared to the control. GBE-associated numerical non-significant trends were found after 6 m for dual task-related gait velocity and stride time variability.	Seven SAEs in four patients in GB group and six SAEs in five patients of control group: nasal septum surgery, diverticula, suspected coronary heart disease, pancreatitis, symptomatic cholecystolithiasis, and transient ischemic attack.
*Gingko biloba* and Diabetes Mellitus					
[29]	Lithuanian	Randomized double-blind placebo-controlled with 56 patients with T2DM (21 male and 35 females; 37–78 y) and followed up for diabetic retinopathy, nephropathy, or neuropathy.	Patients received standardized GB dry extract (80 mg) or placebo capsules. For the first 9 m, patients used one capsule 2 times/d, and for the second 9 m, one capsule 3 times/d.	The level of perceived stress was reduced significantly after 9 m and 18 m, and the psychological aspect of quality of life significantly improved after 18 m of GB use.	AEs were not reported.
[30]	Iraq	Randomized, placebo-controlled, double-blinded, multicenter trial with 60 T2DM patients, 25–65 y.	The patients currently using metformin were allocated to receive GB extract (120 mg/day) or placebo/3 m.	GBE significantly reduced HbA1c, glycemia and insulin levels, BMI, WC, and VAI. GB extract did not negatively impact the liver, kidneys, or hematopoietic functions.	No SAEs were observed.
*Ginkgo biloba* and Metabolic Syndrome					
[31]	Bulgaria	Randomized preventive study with 11 patients (two male, nine female, 26–48 y) with MS, smokers, and Lp (a) concentration > 30 mg/dL.	The standard therapy was EGB 761 120 mg 2 times/d/2 m. No statins, no calcium antagonists, and no nitrate compounds were given.	There was a decrease in oxidative stress biomarkers, atherosclerotic plaque formation, plaque stability and progression, and inflammation.	No AEs occurred.
[32]	Bulgaria	Randomized preventive study with 11 patients (two male, nine female, 26–48 y) with MS, smokers, and Lp (a) concentration > 30 mg/dL.	The standard therapy was EGB 761 120 mg 2 times/d/2 m. No statins, no calcium antagonists, and no nitrate compounds were given.	Simultaneous decreases in hs-CRP and HOMA-IR, as well as a beneficial change in arteriosclerotic, inflammatory, and oxidative stress biomarkers, were observed. IL-6 and nano-plaque formation were additionally reduced.	No AEs occurred.

AD—Alzheimer’s disease; BMI—body mass index; DSM IV—Diagnostic and Statistical Manual of Mental Disorders, 4th edition; GB—*Ginkgo biloba*; GBE—*Ginkgo biloba* extract; EGb 761—extract of *Ginkgo biloba* leaves (drug extract ratio 35–67:1); GBT—*Ginkgo biloba* tablet; HbA1c—glycated hemoglobin; HOMA- IR—homeostasis model assessment of insulin resistance; hs-CRP—high-sensitivity C-reactive protein; IL-6—interleukin 6; Lp (a)—blood lipoprotein(a); MCI—mild cognitive impairment; MMSE—Mini-Mental State Examination; MoCA—Montreal Cognitive Assessment; NPI—Neuropsychiatric Inventory; NPS—neuropsychiatric symptoms; SAEs—serious adverse events; SKT—Short Cognitive Test; SMT—Seven Minute Test; TCD—transcranial Doppler; T2DM—type 2 diabetes mellitus; VAI—visceral adiposity index; VCIND—vascular cognitive impairment of none dementia; WC—waist circumference; d—day; w—week; y—year.

## 3. Results

**Figure 2:**
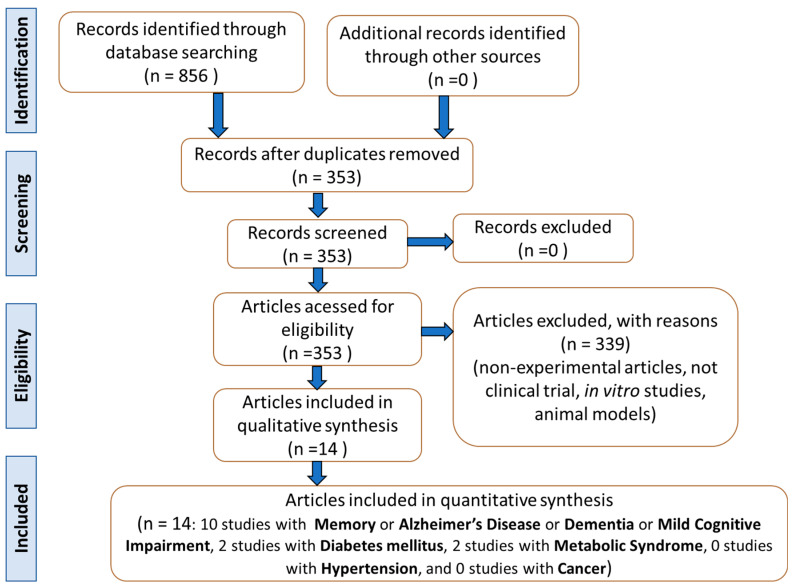
Flow diagram showing the study’s selection criteria (based on PRISMA guidelines).

Figure 2 represents the scheme of the search for studies. From the 14 articles selected, a total of 1681 participants were included: 188 mentally healthy, 410 with mild to moderate dementia, 410 with mild to moderate dementia and neuropsychiatric symptoms, 56 with AD, 80 with vascular cognitive impairment of none dementia, 34 symptomatic irradiated brain tumor survivors, 210 with mild cognitive impairment, 80 cerebral infarction patients, 75 with subjective memory impairment, 116 with type 2 diabetes mellitus, and 22 with metabolic syndrome. Nine hundred eleven participants were women and 472 were men. Two studies did not report the gender of the participants. The age range was over 25 years. No clinical trials that investigated the effects of GB on Parkinson’s disease and cancer were found.

From the 14 articles (three from Germany (Kaschel et al., 2011 [19], Beck et al., 2016 [27], Gschwind et al., 2017 [28]); one from Ukraine (Ihl et al., 2011 [20]); one from the Republic of Belarus, Republic of Moldova, and Russian Federation (Herrschaft et al., 2012 [21]); one from Iran (Nasab, 2012 [22]; two from China (Zhang, 2012 [23], Wang, 2015 [26]); one from EUA (Attia et al., 2012 [24]); one from Russia (Gavrilova, 2015 [25]); one from Lithuania (Lasaite et al., 2015 [29]); one from Iraq [30]); and two from Bulgaria (Siegel et al., 2011 [31], Siegel et al., 2014 [32]), nine were randomized double-blind placebo-controlled clinical trials (Kaschel et al. 2011 [19], Beck et al., 2016 [27], Gschwind et al., 2017 [28], Ihl et al., 2011 [20], Herrschaft et al., 2012 [21], Nasab, 2012 [22], Gavrilova, 2015 [25], Lasaite et al., 2015 [29], Aziz et al., 2018 [30]); one was an open-label phase II clinical trial (Attia et al., 2012 [24]); and four were randomized placebo-controlled clinical trials (Zhang, 2012 [23], Wang, 2015 [26], Siegel et al., 2011 [31], Siegel et al., 2014 [32]).

Seven studies used GB 761 extract (Kaschel et al., 2011 [19], Beck et al., 2016 [27], Ihl et al., 2011 [20], Herrschaft et al., 2012 [21], Gavrilova, 2015 [25], Siegel et al., 2011 [31], Siegel et al., 2014 [32]), two used GB (Nasab, 2012 [22], Attia et al., 2012 [24]); two used GB tablets (Zhang, 2012 [23], Wang, 2015 [26]); and three used GB extract (Gschwind et al., 2017 [28], Lasaite et al., 2015 [29], Aziz et al., 2018 [30]). The administered doses ranged from 40 mg per day to 240 mg per day, and the intervention period ranged from 6 weeks to 36 weeks. Two studies were associated with the use of aspirin (Zhang, 2012 [23], Wang, 2015 [26]) and one study was associated with the use of metformin (Aziz et al., 2018 [30]).

Studies have shown that the use of GB (in different formulations) can improve the memory, cognition, psychopathology, functional measures, and quality of life of patients, in addition to improving cerebral blood flow supply, executive function, attention/concentration, non-verbal memory, and mood and decreasing stress, HbA1c, fasting serum glucose and insulin levels, body mass index, waist circumference, visceral adipose index, biomarkers of oxidative stress, the stability and progression of atherosclerotic plaques, and inflammation. The main adverse effects reported were headache, respiratory tract infection, hypertension, and diarrhea (Table 1).

Table 2 shows the description of the bias in the included studies.

## 4. Discussion

The studies included in this review showed that GB generally and safely improved neuropsychiatric symptoms (SKT, NPI, and MMSE scores), cognition, mood, HbA1C, glycemia, waist circumference, BMI, atherosclerotic lesions formation, pro-inflammatory biomarkers (e.g., IL-6), and quality of life in healthy patients and subjects with mild cognitive impairment or vascular cognitive impairment.

### 4.1. Ginkgo biloba, Inflammation, and Oxidative Stress

Inflammation and oxidative stress are related to the aging process. As a result of the metabolism, several conditions, such as infections, stress, inflammation exposure, radiation, and smoke, produce reactive oxygen species (ROS). When the endogenous antioxidant system or the intake of exogenous antioxidants is insufficient, these molecules can lead to irreversible cell damage and are associated with various diseases, such as diabetes, obesity, hypertension, CVD, cataracts, neurodegenerative diseases, and cancer [33,34,35,36]. Several antioxidants can help to prevent the impact of the aging process. ROS are produced through endogenous and exogenous pathways and can be neutralized by enzymatic and non-enzymatic antioxidants. There are many defense systems, including glutathione peroxidase, catalase, thioredoxin, superoxide dismutase, coenzyme Q, cytochrome c oxidase (complex IV), vitamin E, ascorbic acid, and carotenes [37,38].

GB’s bioactive compounds (Table 3) can act to minimize these conditions, mainly ginkgolide (diterpenoid) A, which is related to the suppression of the cyclo-oxygenase-2 (COX-2) and 5-lipo-oxygenase (5-LOX) enzymes. These molecules are responsible for the conversion of arachidonic acid to leukotrienes, diminishing the inflammatory process. They can reduce the production of malonaldehyde and increase the expression of glutathione (GSH) and superoxide dismutase (SOD). Furthermore, EGB 761^®^ can reduce the effects of the lipopolysaccharide (LPS) and its action on transforming growth factor β (TGF-β), which results in the downregulation of interleukin-1 𝛽 (IL-1 𝛽), IL-6, IL-8, and tumor necrosis factor alpha (TNF-α). On the other hand, ginkgolide B can inactivate platelet-activating factor, which plays a role in the inflammation of the pulmonary airways. In general, ginkgolides A, B, and C decrease ROS levels; the release of TNF𝛼, IL-1𝛽, and IL-6; and the expression of the gene c-fos and the gene c-jun mRNA. They may be related to the inhibition of platelet-activating factor and of the following signaling pathway: NF-kappa-B-inducing kinase (NIK), IκB kinase α (IKK𝛼), nuclear factor kappa-B inhibitor (IkB), and nuclear factor kappa-B-inducing kinase. They are also associated with an increase in cellular proliferation; an increase in the activity of free radical scavengers; and the activation of extracellular signal-regulated kinases, mitogen-activated protein kinase (MAPK) pathways, and hypoxia-inducible factor 1-alpha (HIF-1𝛼) [39,40,41,42].

Kaempferol is also present in GB, and its actions account for the upregulation in the expression of the glutamate-cysteine ligase catalytic subunit, brain-derived neurotrophic factor (BDNF), B-cell lymphoma protein 2 (BCL-2), and GSH; it also reduces serotonin breakdown by monoamine oxidase, the release of cytochrome C, the activity of caspase-3, the downregulation of NFkB, and apoptosis. Kaempferol is also related to the reduction of neurotoxicity induced by 3-nitropropionic acid and the increase in BCL-2-associated protein X through ROS [12,82,83].

Other relevant compounds found in GB are quercetin, bilobalide, and isorhamnetin [67], which also play an important role in inflammation and oxidative stress. Quercetin can promote the elevation of BDNF levels and reduce apoptosis, the transcription of TNF𝛼, the degradation of serotonin by monoamine oxidases, phosphorylation, and the activation of c-Jun *N*-terminal kinase. Moreover, it can act as a free radical scavenger. Bilobalide has actions including the reduction of ROS induced through hydrogen peroxide_._ It is related to the upregulation of BCL-2 and the cytochrome c oxidase subunit III and increases the cellular proliferation of hippocampal neurons. Isorhamnetin is associated with the reduction of apoptosis and the fragmentation of DNA. Indeed, it also reduces the synthesis of pro-inflammatory cytokines and caspase-3. Some studies have shown that isorhamnetin has beneficial effects on the cardiovascular and cerebrovascular system and can have anti-inflammatory, antioxidant, and anti-tumor functions. These effects are associated with the regulation of NFkB, MAPK, PI3K, AKT, and PKB [13,84,85].

Although each bioactive compound has numerous effects on aging-related cellular and metabolic events, EGB 761^®^ plays several critical effects in this process. It can decrease the levels of anion superoxide radical, hydrogen peroxide radicals, ROS and RNS (reactive nitrogen species), peroxyl radicals (ROO), and hydroxyl radicals (OH). In neurology, this plant extract can be used to improve circulation since it protects the cortical neurons from iron injuries and reduces the peroxide levels in cerebellar neurons. Moreover, it can upregulate the expression of antioxidant enzymes, such as glutathione peroxidase and superoxide dismutase [67,86,87,88].

### 4.2. Gingko biloba, Mitochondrial Dysfunction, and Apoptosis

Mitochondria are the organelle responsible for energy production in our cells and can use O_2_ and glucose to produce ATP, CO_2_, and H_2_O. Mitochondrial dysfunction is associated with ROS production that triggers peroxidative reactions, culminating with harmful effects on mitochondrial biomolecules. This impairment in mitochondrial function can lead to neuronal cell death and augmented tissue loss. GB can reduce ROS levels in mitochondria, and EGB 761^®^ can stabilize mitochondrial function. Furthermore, it can protect respiratory chain complexes I, IV, and V in mitochondria and improve mitochondrial membrane potential and morphology linked to aging in the liver and brain.

Interestingly, it can prevent mitochondrial dysfunction in both young and old mice. Still, the protective effect can only be observed in aged animals, possibly because of the increase in the permeability of the brain–blood barrier with aging. GB can also protect and upregulate mitochondrial DNA [89,90,91,92].

EGB 761^®^ is also associated with the inhibition of cytochrome c oxidase activation and the reduction of mitochondrial ATP and GSH with aging. It also regulates the expression of Bas and pBcl-xL and their inhibition of the activation of caspase-9 to protect against mitochondrial dysfunction in rat cochlear tissue [92].

The increase in ROS production is associated with apoptosis, which has a critical role in the aging process, and bilobalide can prevent this process in aged animals. Some authors have demonstrated the use of hydrogen peroxide to induce apoptosis and the utilization of Aβ protein 1–42 to mimic impairments in age-related neurological functions. Bilobalide can inhibit hydrogen peroxide cell apoptosis due to the restriction of mitochondria-mediated caspase activation [89,93].

As already mentioned, EGB 761^®^ can also inhibit the activation of NFκB stimulated by β-amyloid peptide, suppressing the expression of Toll-like receptors and, thus, reducing apoptosis in neuronal cells. NFκB is a critical regulator of cell death programming via apoptosis and necrosis. It is related to proapoptotic gene upregulation, for example, the death receptor Fas and TNF-α. In normal tissues, bilobalide suppresses apoptosis; however, it has an opposite action in cancer cells, inducing apoptosis [94,95].

### 4.3. Ginkgo biloba and Neurodegenerative Diseases

**Figure 3:**
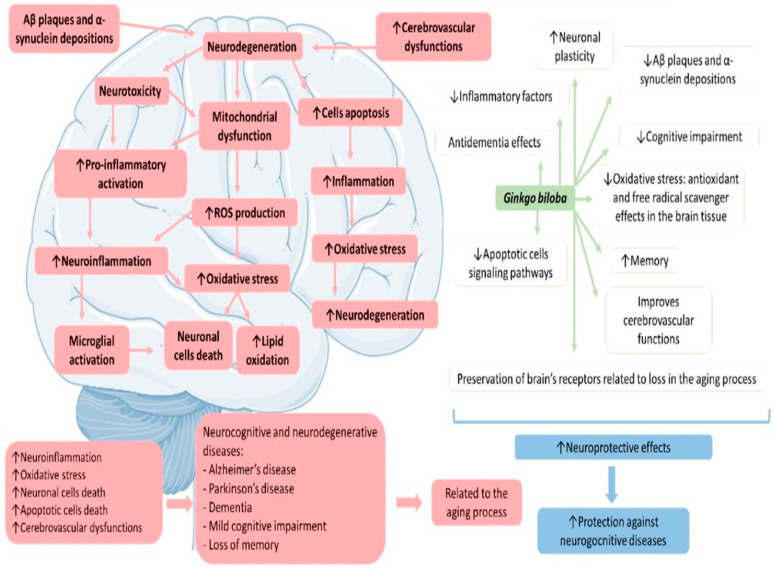
*Ginkgo biloba*: general effects against neurodegenerative diseases. ↑—increase; ↓—decrease; Aβ—beta amyloid.

Neurodegenerative diseases are the leading cause of disabilities in the elderly. Many studies have shown that mitochondrial dysfunction, oxidative stress, neuroinflammation, and apoptosis accompanying the aging process are linked to neurodegenerative conditions [5]. GB possesses twenty-seven active compounds with multi-target synergistic actions for the therapeutic approach to neurodegenerative disorders. These compounds may interfere with biological events, such as the activation of transcription factor activities and oxidative reactions. Moreover, these active compounds can interfere with more than one hundred metabolic pathways [96]. Figure 3 shows the main effects of GB on neurodegenerative diseases.

#### 4.3.1. *Ginkgo biloba* and Memory

The clinical concepts of memory divide this phenomenon into episodic, semantic, working, and procedural aspects. Loss of memory is one of the most common first symptoms of AD dementia, affecting 30 million people worldwide. Although AD is the most well-known type of memory impairment, many different neuro-pathologies can affect the neuronal networks of dissociable memory systems and cause memory loss (principally when the individual experiencing this loss belongs to a group with risk factors). GB extracts are related to the enhancement of cognitive functions, specifically memory, in addition to concentration. The extracts of this plant are the most related to memory improvements via their neuroprotective effects studied in human clinical trials. Since GB presents anti-inflammatory, antioxidant, and antiapoptotic actions, it leads to antidementia environments, together with the regulation of neurotransmitters (such as serotonin) and the expression of neurotransmitter receptors in the human brain. GB is also related to modulations of synaptic plasticity in humans and it regulates structural changes and neurogenesis in hippocampus circuity, affecting neuron excitability [97,98,99].

One study investigated the effects of EGb 761^®^ on memory and the specificity of these effects on distinct memory functions. The results showed that EGb 761^®^ (240 mg once daily) could significantly improve the number of appointments correctly remembered by healthy middle-aged people. This study adds to the evidence that GB can improve memory. However, we observed bias in this study; for example, no patient’s predictive data or demographic characteristics were reported [19] (Table 1).

#### 4.3.2. *Ginkgo biloba* and Dementia

Dementia is a neuronal condition that is increasing in prevalence in the aging population at a tremendous rate, such that 6% of people older than 65 years have some spectrum of dementia. It causes memory loss, followed by reduced executive functions, other cognitive deficits, and changes in the individual’s personality. Initially, individuals with dementia present with a loss of recent events’ memories, and over time, they start to become unable to make decisions and sequence complex tasks. There are many types of dementia, but the most common forms present one similar pathophysiological feature: cerebrovascular dysfunction. Many factors are involved in the dysfunction of the central nervous system’s circulation and its relation to the pathophysiology of dementia. Cerebrovascular alterations and the apogee of dementia are associated with hypoxia, hypoperfusion, and dysfunctions in cerebrovascular hemodynamics [100,101,102].

Hypoxia and hypoperfusion lead principally to decreased cerebral blood flow and the occurrence of micro-infarcts and white matter abnormalities in brain tissue. More serious ischemic events in the brain tissue can also be associated with dementia. Alterations in the cerebrovascular hemodynamics are related to the impairment of cognitive functions that occur due to endothelial damage, changes in the neurovascular microvascular anatomy, modifications in vascular remodeling, neurovascular reactivity damage (blood vessel tortuosity and vessel-wall thickening), increases in oxidative stress, and increases in blood pressure. The emergence of dementia can be related to metabolic dysfunctions, such as impaired glucose metabolism and mitochondrial dysfunctions. The cerebrovascular and metabolic dysfunctions lead to neuroinflammation, and synaptic loss and neurodegeneration also occur. The brain’s atrophy and the alterations in the permeability of the blood–brain barrier are related to the different causes of dementia [9,101,103].

GB can be used to treat and prevent dementia since it exhibits neuroprotective effects. It can protect against neuronal death by ischemic events and is associated with improvements in blood circulation by the reinforcement of capillary walls, preventing neuronal cell harm by hypoxia. GB extracts are also related to neuroprotection and improvements in neuronal plasticity. In vitro studies have demonstrated that they can protect neuron cultures against the harmful effects of hydrogen peroxide. GB can also improve memory implications and preserve the brain through the aging process, principally by protecting neuronal cells’ receptors related to age loss in the aging process, which can be associated with the counteractions of cognitive impairments [9,28,101,102,104].

In a randomized, double-blind, multicenter study with a significant number of participants and an adequate follow-up, the authors showed that a once-daily formulation of EGb 761^®^ in the treatment of dementia in patients with neuropsychiatric features was safe and superior to the use of a placebo in this population [20]. Another multicenter, double-blind, randomized, placebo-controlled trial was conducted to demonstrate the efficacy and safety of EGB 761^®^ extract in patients with mild to moderate dementia associated with neuropsychiatric symptoms. The primary outcomes were changes from baseline to week 24 in SKT and NPI total scores. The Verbal Fluency Test, ADCS Clinical Global Impression of Change (ADCS-CGIC), International Activities of Daily Living Scale (ADL-IS), DEMQOL-Proxy quality of life scale, and 11-point box scales for tinnitus and dizziness were used as secondary outcome measures (Table 1) [21].

#### 4.3.3. *Ginkgo biloba* and Mild Cognitive Impairment

MCI is characterized as a neurocognitive state of subjective complaints of impairments in an individual’s cognitive performance. It is understood to be a mild cognitive state between normal cognitive aging and dementia. Although there are many diagnostic criteria for MCI, it is recognized that this neurocognitive condition corresponds to objective evidence in the lack of dementia diagnostic criteria in a subject. It is not related to a specific etiology. Still, it can be an early manifestation of AD (similar to a prodromal stage) or even a risk factor for this disease and other neurodegenerative conditions. The risk factors for this neurological condition include being of the male sex and older age. However, it is known that in older people, the principal risk factors for the development of MCI go beyond the traditional: depression, polypharmacy, and uncontrolled CVD. CVD has also been demonstrated to be a risk factor for the progression of MCI to AD. The prevalence of MCI increases by the age of 65 years, and it is known that the affected subjects can progress to dementia, remain at the MCI stage, or regress to normal. Although the identification and classification of MCI are considered to be a significant challenge, the diagnosis of this condition comprises neuroimaging, clinical assessments, and a neurophysiological evaluation. No medications are considered effective in combating dementia, mainly because MCI patients are only steps away from having dementia [105,106,107].

MCI subjects can demonstrate both neurocognitive and neuropsychiatric symptoms. The most common neurocognitive symptoms are impairments and alterations that lead to abnormalities in complex attention, social cognition, memory, learning, language function, perceptual-motor function, and executive function. Besides that, the neuropsychiatric symptoms may be summarized by changes in personality or usual conduct, depression, apathy, irritability, sleep and appetite disturbances, dysphoria, and hallucinations or delusions [25,107,108].

GB and its extracts show beneficial effects on cognitive dysfunctions, CVD, in the treatment of MCI by improving memory, learning abilities, and executive functions. GB and its derivatives enhance neuronal plasticity and mitochondrial function, promote neurogenesis, and improve neuronal energy metabolism. Besides that, GB can affect the neurotransmitter levels in the brain and has actions on the microcirculation and the brain’s micro-perfusion. All of these effects can be associated with ameliorations in memory and, consequently, in MCI and MCI progression [25,108,109].

Gait instability in MCI patients, particularly in dual-task situations, has been associated with impaired executive function and an increased risk of falls. GB extract can be effective in improving gait stability [28]. In a study that associated 75 mg aspirin to 19.2 mg GB for the treatment of cognitive vascular impairment of non-dementia after three months, MoCA scores for executive ability, attention, abstract, delayed memory, and orientation were significantly increased compared to those before treatment and with the controls after treatment. Furthermore, the blood flow velocity of the anterior cerebral artery was significantly augmented. However, the study did not present demographic data, nor randomization or blinding data, nor the results on adverse effects; in addition, it used a small dose of GB when compared to other studies (Table 1) [23]

An open-label phase II study was conducted to assess the effects of GB in symptomatic irradiated brain tumor survivors. GB improved the patients’ quality of life and cognitive function. However, a high dropout rate and a small sample may have interfered with the results in this study [24]. Another study showed the beneficial effects of EGB 761^®^ on neuropsychiatric symptoms (NPS) and cognition in patients with MCI. It was observed to ameliorate NPS and cognitive performance in MCI patients, which are related to faster cognitive decline and an increased risk of developing AD. As EGb 761^®^ is safe and well tolerated, it represents a promising treatment option for MCI as defined by international consensus criteria (Table 1) [25].

A study by Wang et al. also linked 75 mg aspirin to 40 mg GBT for the treatment of vascular cognitive impairment of non-dementia and demonstrated that, in general, GB could be used to improve cerebral blood flow and cognitive ability in patients with this condition. However, the study did not present diverse data, such as demographic data, randomization data, blinding data, or adverse effects. In addition to using a small dose of GB, the authors also specify in their abstract that they used 19.2 mg of GBT thrice a day; however, in the methods section, the authors indicate that 40 mg were used three times a day in the combined treatment group [26]. Moreover, this trial seems to be the same as Zhang et al. (2012), yet this study was not mentioned (Table 1).

One study found indications for improved cognitive flexibility without changes in brain activation, suggesting increased processing efficiency with EGb761^®^, along with a trend towards better response inhibition results compatible with a slight increase in prefrontal dopamine. Although these conclusions must be confirmed, EGb761^®^ was shown to be safe and well tolerated. However, the study did not show demographic data, had a significant sample loss during the investigation, and did not perform a sample calculation (Table 1) [27].

#### 4.3.4. *Ginkgo biloba* and Alzheimer’s Disease

AD is a condition that accounts for one of the most distinguished global healthcare issues and is the third leading cause of death in the United States. The etiology of this disorder is not completely understood, but genetic factors are linked to approximately 10% of cases. The available therapies cannot cure AD and, in many cases, show limited effectiveness in the treatment of AD [110,111,112].

The pathophysiological processes triggered in this disease involve neuronal degeneration and the waste of synapses in the cortex, hippocampus, and subcortical areas, resulting in atrophy, loss of memory, executive dysfunction, mood swings, and an inability to learn new information and perform daily living activities [113,114]. The neurodegeneration that occurs in AD is associated with the elevation in the levels of Aβ42, an altered form of the amyloid-β peptide. This aberrant Aβ42 results in the production of extracellular oligomers and aggregates and leads to the hyperphosphorylation of the tau protein, culminating with deposition as insoluble neurofibrillary tangles. These processes interfere with synaptic function and neuronal survival. Moreover, glial cells also become abnormal, contributing to the pathophysiology of the disease [115,116].

When there is an accumulation of extracellular Aβ plaques, there is stimulation of astrocytes and microglia, resulting in the release of pro-inflammatory cytokines. The chronic release of these molecules leads to neuroinflammation, which is conducive to synapse loss and neuronal death. The imbalance in the functions of microglia and astrocytes is also related to the augmentation of extracellular glutamate, which is related to the neuron excitotoxicity resulting from the overactivation of the *N*-methyl-d-aspartate receptors (NMDA). Besides that, in the neuroinflammation scenario, astrocytes and microglia lose their capacity to release cytokines related to neuron survival and functioning [112,117,118,119].

The failure of available drugs targeting β-amyloid and tau proteins suggests a need for other preventative and therapeutic strategies for AD [120]. GB exhibits anti-inflammatory, antioxidant, and antiapoptotic actions; for these reasons, it can stimulate neurogenesis and cerebral blood flow, improve mitochondrial and neuronal function, and inhibit neural cell death. Beyond that, GB has anti-platelet-activating factor actions in vascular conditions, inhibits β-amyloid aggregation, and reduces the peripheral benzodiazepine receptor expression for stress relief. In vitro, it can reverse β-amyloid and NO-induced toxicity and diminish apoptosis. GB can also work as an iron-chelating compound that can also inhibit the formation of Aβ fibrils. It can also play a role as a cholinesterase inhibitor and delay the progression of the disease. Further, the use of GB is associated with mild or no side effects and can improve the quality of life in AD patients [10,93,121,122,123,124].

As mentioned before, the protective effects of GB against Aβ-induced neurotoxicity occur through the inhibition of Aβ-induced events, such as the accumulation of ROS; glucose uptake; mitochondrial dysfunction; the activation of JNK, ERK, and AKT pathways; and apoptosis. It can also inhibit the synthesis of Aβ in the brain by reducing circulating free cholesterol (amyloidogenesis); AβPP processing is potentially affected by the levels of free circulating and intracellular cholesterol [9,125,126,127].

Other properties of GB and GB extract reside in the improvement of blood circulation and the protection of the capillary walls and nerve cells from damage when oxygen is devoid. It can also be considered in the treatment of concentration disorders, memory impairment, and dementia. It shows positive effects on neurological and cognitive functions since it regulates vascular flow. Apart from its free radical scavenger property, GB also interferes in the transcription of many genes linked to oxidative stress, protecting the neuronal cells against the harmful effects of ROS [9,120,128].

Nasab et al. [22] compared rivastigmine, a cholinesterase inhibitor, with GB for dementia (AD type), and suggested that the drug is more effective than GB in treating Alzheimer’s dementia (Table 1).

### 4.4. Ginkgo biloba, Metabolic Syndrome and Cardiovascular Diseases

Metabolic syndrome (MS) is one of the leading public health problems today for men and women (reaching almost 30% in some populations). It is defined for different diagnosis criteria, including cardiometabolic risk factors, such as insulin resistance, high triglycerides levels, low HDL-c levels, obesity (augmented waist circumference), and hypertension. An individual is considered to possess MS when presenting at least three of these risk factors. In this scenario, a pro-inflammatory state should also be considered in patients with MS, and chronic inflammatory conditions are related to the rise in the occurrence of CVD [129,130].

GB extract may have a significant antidiabetic effect. It can expand glycogen levels in the muscle and liver and thus can decrease plasma glucose levels. Moreover, it can reduce HbA1c, insulin levels, body weight, waist circumference, and visceral adiposity index. Priyanka et al. [131] and An et al. [132] have suggested that GB can improve insulin resistance and inflammation resulting from the increase in the secretion of adiponectin, reducing serine phosphorylation of IRS-1 receptors, reducing NFκB/JNK activation, and, consequently, reducing the release of inflammatory adipokines.

**Figure 4:**
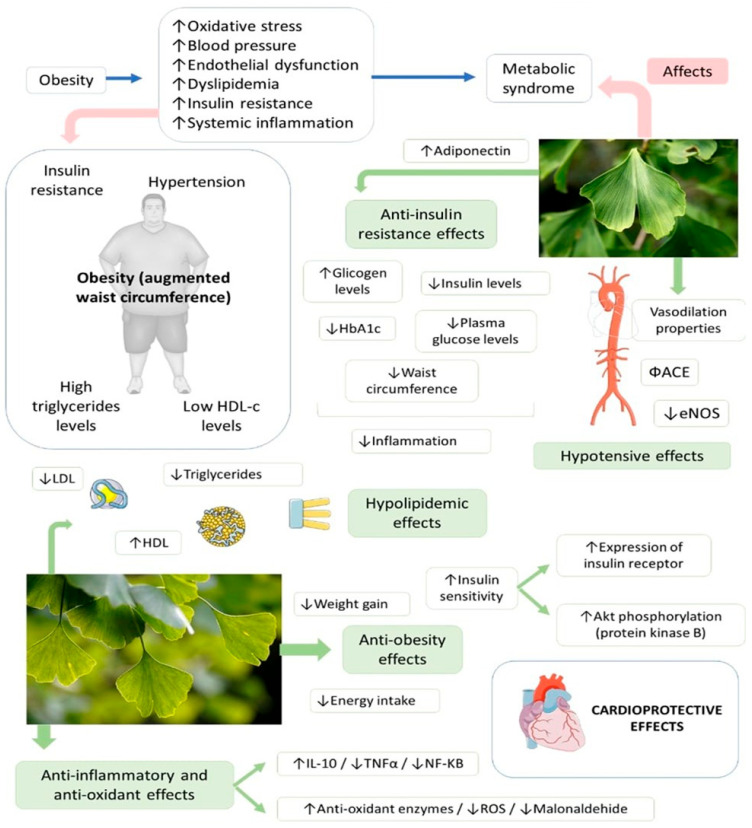
*Ginkgo biloba* and its extracts have many effects against cardiovascular risk factors that compound metabolic syndrome. ↑—increase; ↓—decrease; Φ—inhibition; ROS—reactive oxygen species; TNF-α—tumor necrosis factor; IL-10—interleukin 10; NK-KB—factor nuclear kappa B; ACE—angiotensin-converting enzyme; eNOS—nitric oxide synthase 3.

The use of GB has also been shown to be effective in the reduction of cholesterol absorption in rats; in the inhibition of 3-hydroxy-3-methylglutaryl–coenzyme A, an enzyme that is a center of regulation of cholesterol synthesis; and in the improvement of high-fat diet-induced hyperglycemia [133,134]. In rabbits, GB significantly diminished triglycerides and cholesterol levels and increased HDL-c. Besides that, GB increased the levels of antioxidant enzymes and decreased malonaldehyde levels [135]. GB extract can also reduce body weight and weight gain, and can upregulate the expression of IL-10 (and downregulate the expression of TNF-α and NFκB), insulin receptor (IR), and protein kinase B (Akt) phosphorylation, stimulating the insulin signaling cascade [136]. GB also has hypotensive actions related to its capacity for angiotensin-converting enzyme (ACE) inhibition and vasodilation, and its ability to increase the expression of endothelial nitric oxide synthase (eNOS) [8,137]. Figure 4 shows the effects of GB on MS.

GB was also associated with an improvement in cardiomyopathy, which is a common reason for heart failure and can lead to a higher risk of cardiac death. Due to its unclear pathogenesis, cardiomyopathy lacks an effective treatment, and new strategies are required. The beneficial effects of GB and its bioactive compounds in this pathological condition are linked to the improvement of blood circulation and other multi-pathways associated with the regulation of antiapoptotic, pro-survival, and anti-inflammatory actions via NFκB and PI3K-AKT signaling [138,139].

Aging-related vascular pathology is closely linked to endothelial dysfunction and arterial stiffening that culminate in CVD progression. As already mentioned in this paper, oxidative stress and inflammation lead to vascular impairment. The antioxidant and anti-inflammatory actions of GB extract are related to amelioration of aging-related vascular impairment. The main activities of this plant in aged vasculature are probably linked to the longevity signaling pathways and the slowing of vascular aging progression in diabetes owing to the regulation of glycemia and lipid metabolism [140,141].

**Figure 5:**
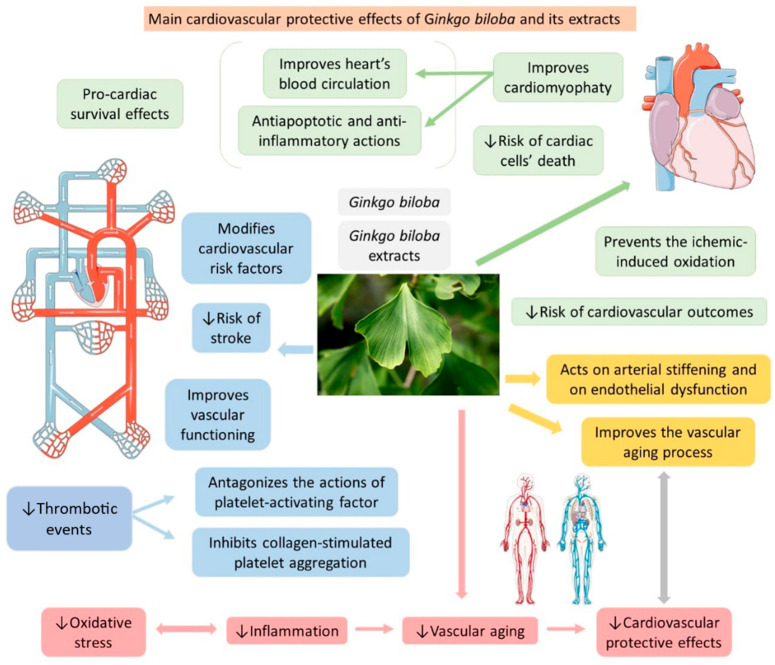
*Ginkgo biloba* and its extracts have cardiovascular protective effects that improve the functionality of the cardiovascular organs. ↓—decrease.

Moreover, several studies have suggested that ginkgolide A plays a role as an antithrombotic agent and could be used for prevention and/or for controlling thrombosis. It can inhibit platelet aggregation and collagen-stimulated platelet aggregation due to the activation of MMP-9 and the intracellular production of cAMP and cGMP, which inhibits the mobilization of intracellular Ca^2+^ and reduces the release of thromboxane A2 by inhibiting COX-1 [142,143]. Figure 5 shows some effects of GB on CVD.

A survey in Lithuania aimed to assess the glycemic control and psychological status of patients’ with type 2 diabetes mellitus (T2DM) after antioxidant plant preparations. Patients received a standardized dry extract of GB leaves, green tea dry extract, or placebo capsules. Glycemic control, HbA1c, antioxidant status, and psychological parameters were evaluated at baseline, and after nine and eighteen months of using antioxidant preparations or a placebo. GB leaf extract exhibited a moderate effect on psychological status and a tendency to improve glycemic control in patients with T2DM (Table 1).

One study determined that GB extract as an adjuvant effectively improves metformin treatment outcomes in T2DM patients. However, the main limitations of this study include its small sample size, relatively short duration, and lack of dose-response data for GB extract as an adjuvant to the antidiabetic drug. For these reasons, further studies are necessary to determine the long-term effects of GB extract with a larger sample [30,42].

Siegel, Ermilov et al. [31] suggested that GB may be used as a complementary drug with a preventive character after a percutaneous intervention stent implantation and myocardial revascularization graft in patients with MS (Table 1). Moreover, in another publication with the same sample, Siegel et al. [32] showed that GB could reduce CVD risk factors since it reduces HOMA-IR, hs-C reactive protein, and IL-6.

### 4.5. Ginkgo biloba Bioavailability and Safety

A study investigating the absorption of radiolabeled GBE in animals showed a minimum absorption of 60%. Thus, GB extract is well tolerated and safe. Acute toxicity studies showed a lethal dose (LD50) of 1100, 1900, and 7700 mg/kg in mice and 1100, 2100, and over 10,000 mg/kg in rats when administered intravenously, intraperitoneal, and orally, respectively. The extensive use of GBE in the elderly population with T2DM, hypertension, or rheumatism, can interact with simultaneous drugs. Furthermore, GB supplements are associated with prolonged bleeding times in patients and are contraindicated during pregnancy or breastfeeding [20,21,144,145].

To the best of our knowledge, this is the first review showing the effects of *Ginkgo biloba* in the aging process.

### 4.6. Implication and Limitations

Most chronic degenerative diseases (including those related to aging) are related to oxidative stress and inflammatory aspects. Thus, GB could work as a complementary medicine in several aspects of these diseases.

On the other hand, this review has several limitations, such as the heterogeneity of the outcomes of the included studies, the different formulations of GB, doses used, and the age of the patients in the different studies. Moreover, only English studies were included, and the descriptive review had less evidence than systematic review.

## 5. Conclusions

This review showed that GB could be considered in the therapeutic and preventative approaches to aging-related conditions, such as neurodegenerative disorders, metabolic syndrome, and cardiovascular diseases. From this perspective, GB can be beneficial in chronic degenerative conditions associated with the aging process. Nevertheless, the existing clinical trials are heterogeneous since the different formulations, dosages, and administration times were variable. For these reasons, other studies are necessary to establish the doses, pharmaceutical form, and treatment time needed for preventive effects or therapeutic adjuvants in aging conditions.

## Figures and Tables

**Table 2:** Descriptive table showing the biases of the included randomized clinical trials.

Study	Question Focus	Appropriate Randomization	Allocation Blinding	Double-Blind	Losses (<20%)	Prognostics or Demographic Characteristics	Outcomes	Intention to Treat Analysis	Sample Calculation	Adequate Follow-Up
[19]	Yes	Yes	Yes	Yes	Yes	No	Yes	NR	Yes	Yes
[20]	Yes	Yes	Yes	Yes	Yes	Yes	Yes	Yes	Yes	Yes
[21]	Yes	Yes	Yes	Yes	Yes	Yes	Yes	Yes	Yes	Yes
[22]	Yes	NR	Yes	Yes	Yes	Yes	Yes	No	NR	Yes
[23]	Yes	NR	NR	No	NR	No	Yes	No	NR	Yes
[24]	Yes	NR	No	No	No	Yes	Yes	No	Yes	Yes
[25]	Yes	Yes	Yes	Yes	Yes	Yes	Yes	Yes	Yes	Yes
[26]	Yes	NR	NR	No	NR	No	Yes	No	NR	Yes
[27]	Yes	Yes	Yes	Yes	Yes	No	Yes	No	No	Yes
[28]	Yes	Yes	Yes	Yes	Yes	Yes	Yes	No	Yes	Yes

NR—not reported.

**Table 3:** Bioactive compounds of *Gingko biloba* and their effects on aging-related conditions.

Bioactive Compound	Sources	Molecular Structure	Functions	References
**Ginkgolide A** **(terpenoid)**	Leaves, root, and bark.	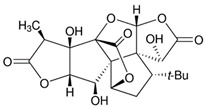	- Anti-inflammatory (decreasing TNF-α, IL-1β, and NF-kB expression); - Antioxidant (reducing ROS and augmenting free radical capture by the cells); - Anxiolytic-like effects; - Neuroprotection (controlling neurodegeneration and inflammation); - Anti-atherosclerotic (prevention of OS to the endothelial cells/stimulation of NO); - Anti-thrombotic (inhibition of platelet aggregation by MMP-9 and controlling cAMP, inhibiting intracellular Ca^2+^ mobilization, and decreasing TXA2 activity); - Hepatoprotective (suppressing hepatocyte lipogenesis); - Antitumor (inhibition of cancer cell proliferation).	[12,42,43,44]
**Ginkgolide B** **(terpenoid)**	Leaves, root, and bark.	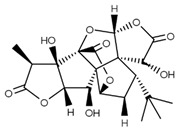	- Neuroprotective effects (protecting neurons from βA apoptotic events and in ischemia/reperfusion syndrome through the regulation of NF-kB pathways); - Anti-inflammatory (decreasing TNF-α, IL-1β, and NF-kB expressions); - Antioxidant (reducing ROS and augmenting free radical capture); - Protective effects of cardiomyocytes against ischemia/reperfusion syndrome; - Inhibition of cancer cell migration and invasion; - Induction of cancer cell apoptosis.	[12,43,45,46,47,48,49,50,51]
**Ginkgolide C** **(terpenoids)**	Leaves, root, and bark.	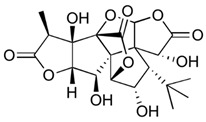	- Anti-inflammatory (decrease in TNF-α, IL-1β, and NF-kB expression); - Antioxidant (reduces ROS and augments free radical capture); - Suppressor of adipogenesis via AMPK signaling pathways; - Hepatoprotective by protecting liver from lipid accumulation injuries; - Alleviation of ischemia/reperfusion syndrome in cardiomyocytes; - Antitumor effects (cancer cells apoptosis and inhibition of cancer cell growth).	[12,43,52,53,54,55,56]
**Bilobalide** **(terpenoid)**	Leaves and bark.	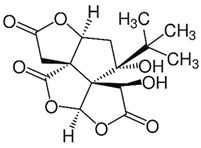	- Anti-inflammatory (decrease in TNF-α, IL-1β, and IL-6 levels); - Neuroprotective (reduction in neuroinflammation and protection against βA deposition in AD); - Hepatoprotective; - Antioxidant via multiple pathways; - Cardioprotective.	[12,43,57,58,59]
**Ginkgolic acid** **(organic acid)**	Leaves.	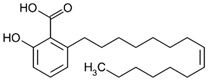	- Antibacterial and antiviral (suppression of gram-positive bacteria growth and fusion of enveloped viruses); - Antitumor effects (inhibiting invasion and migration of cancer cells).	[43,60,61,62]
**Isorhamnetin** **(flavonoid)**	Leaves.	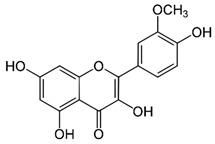	- Anti-atherosclerosis and endothelium protective; - Neuroprotection (improvement of brain function and cognition); - Hypotensive effects; - Anti-ischemia and anti-fibrosis in myocardium; - Anti-inflammatory/antioxidant, - Antitumor effects (suppression of cancer growth and invasiveness).	[43,63,64,65,66,67]
**Quercetin** **(flavonoid)**	Leaves.	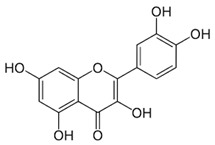	- Anti-inflammatory/antioxidant (decrease in lipid peroxidation and OS); - Increase in BDNF; - Reduces the degradation of serotonin by monoamine oxidases; - Antitumor (modulation of VEGF, P13K/Akt, apoptosis, mTOR, MAPK/ERK1-2, and Wnt/β-catenin signaling pathways); - Attenuation of atherosclerotic inflammation; - Cardioprotection (protection against OS/improvement of cardiomyocytes); - Antimicrobial.	[43,67,68,69,70,71,72,73]
**Kaempferol** **(flavonoid)**	Leaves.	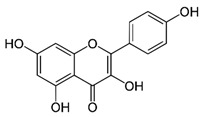	- Antitumor (inhibiting cancer cell proliferation and stimulating apoptosis); - Antioxidant (upregulation of GSH); - Anti-inflammatory (inhibiting NF-kB, COX-2, and iNOS expression); - Neuroprotection (suppression of oxidative and inflammatory damage to brain cells); - Protection against ischemia/reperfusion syndrome and myocardial injury; - Upregulation of BDNF; - Reduction of serotonin degradation.	[12,43,74,75,76,77,78]
**Luteolin** **(flavonoid)**	Leaves.	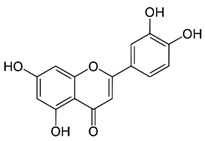	- Anti-inflammatory (suppressing TNF-α, IL-6, COX-2, and NF-kB expressions); - Antioxidant; - Antitumor (inhibiting cancer cell proliferation and stimulating cell cycle arrest and apoptosis); - Neuroprotective (limiting βA deposition, reducing neuroinflammation and brain OS); - Cardioprotective effects (stimulation of cardiomyocyte function through MAPKs); - Reduction of cardiomyocyte ischemic/reperfusion syndrome.	[43,79,80,81]

AD—Alzheimer’s disease; AMPK—AMP (adenosine monophosphate)-activated protein kinase; βA—beta amyloid; BDNF—brain-derived neurotrophic factor; Ca—calcium; cAMP—cyclic adenosine monophosphate; COX-2—cyclooxygenase 2; GSH—glutathione; IL-1β—interleukin 1 beta; iNOS—nitric oxide synthase; IL-6—interleukin 6; MMP-9—matrix metallopeptidase 9; mTOR—mammalian target of rapamycin; MAPK/ERK1-2—mitogen activated protein kinase/extracellular signal-regulated kinase 1-2; NO—nitric oxide; NF-kB—nuclear factor kappa b; OS—oxidative stress; P13K/Akt—phosphatidyl inositol-3-kinase/protein-kinase b; ROS—reactive oxygen species; TXA2—thromboxane A2; VEGF—vascular endothelial growth factor.
